# A Case of Monstrosity

**Published:** 1868-09-01

**Authors:** R. W. Long

**Affiliations:** New Maysville


### A CASE OF MONSTROSITY.

New Maysville, *June 25th*, 1868.

Prof. Allen—Dear Sir: Allow me to refer to you a case of monstrosity which fell into my hands not long ago. April 26, 1868, I was summoned to attend Mrs. L., aged 36 years, in her ninth confinement—eight months advanced. She was seized at 7 A.M. with violent pains, which were followed by the escape of the liquor amnii—nearly half a gallon, according to her statement, came away. The pains ceased to recur until afternoon. I proceeded to make an examination: found the os uteri dilated about the size of a quarter of a dollar, and the membranes pouching through, and could barely touch what I took to be an elbow. I was unable to make out a positive diagnosis, as it would recede from my finger on the slightest touch. After the elapse of several hours, the os was sufficiently dilated so that I ruptured the membrane, and near a quart escaped. I introduced my hand and found that I had a knee, instead of an elbow presented. I succeeded in bringing down the feet, and congratulated myself with the thought that delivery would soon take place; but pain after pain caused but little or no progress. I gave her *Ergot.* The contractions became very active. I made gentle but firm traction, but ineffectually. Her powers began to fail. I thought I was justifiable in a stronger effort, which was made, and I soon discovered I was making some progress. In examining the breach, I could feel no trace of any genital organs, but distinctly felt a tumor on the left hip nearly as large as a pullet's egg, soft and compressible. After delivering the breach, I felt something in or protruding through the vagina, which I first thought to be the placenta, but another firm contraction, and I felt quite a different substance. On examination the first proved to be the liver of the child, the remainder was the abdominal viscera, and that the child was neither male nor female, there being no trace of the genitals or rectum. The abdomen was contracted, and the viscera *developed on the outside*, surrounded by the membrane, which I had previously ruptured. The opening was not partially, but perfectly closed. They were attached by a small place (one-half inch in diameter). Just below the umbilicus and the umbilical cord, or what served its purpose, was a small, flat substance, about one-fourth the normal size, and two and one-third or three inches long, and seemed to unite or be continuous with the membranes covering the intestines. There was some little deformity of the lower extremities, but the head, thorax, and upper extremities were well developed. I think the shortness of the cord was the cause of the protracted labor, which gave way at the last effort of traction. After delivery, the most frightful hæmorrhage ensued. I introduced my hand and delivered the placenta, and had no farther trouble. Yours, truly,

R. W. LONG, M.D.

